# Executive functions assessment in patients with language impairment A systematic review

**DOI:** 10.1590/1980-57642018dn12-030008

**Published:** 2018

**Authors:** Ana Paula Bresolin Gonçalves, Clarissa Mello, Andressa Hermes Pereira, Perrine Ferré, Rochele Paz Fonseca, Yves Joanette

**Affiliations:** 1 Psychology Graduate Student, Pontifícia Universidade Católica do Rio Grande do Sul, RS, Brazil.; 2 Master student, Universidade Federal do Rio Grande do Sul, RS, Brazil.; 3 PhD, Centre de Recherche de l’Institut Universitaire de Gériatrie de Montréal;; 4 PhD, Pontifícia Universidade Católica do Rio Grande do Sul, RS, Brazil.

**Keywords:** executive functions, acquired language impairment, neuropsychological assessment, funções executivas, distúrbios de linguagem adquiridos, avaliação neuropsicológica

## Abstract

Acquired language impairments may accompany different conditions. Most recent studies have shown that there is an important relationship between language and cognitive functions, such as executive functions (EF). Therefore, we aimed to investigate which main EF components appear to have the greatest impact in the most prevalent acquired communication disorders in adults, and which neuropsychological tests are being used to evaluate them. In addition, we sought to characterize the relationship between the executive functions and language in these conditions. Working memory (WM) was the most frequently chosen cognitive measure, being evaluated by different span tasks. A relationship between WM and narrative and conversational discourse, writing abilities and grammatical comprehension was found. Other currently used cognitive tests included the Trail Making, Wisconsin, Stroop and Verbal Fluency tests. Language and EF have a complex relationship; hence, a complete assessment should reflect the dynamic processing of cognitive brain functions.

## Resumo

Distúrbios de linguagem adquiridas podem acompanhar diferentes quadros. Estudos recentes mostram que existe uma relação importante entre linguagem e funções cognitivas, como as funções executivas (FE). Portanto, visou-se investigar quais os principais componentes das FE parecem ter um maior impacto nos quadros com transtornos de comunicação adquiridos mais prevalentes em adultos e quais testes neuropsicológicos estão sendo mais utilizados para avaliá-los. Além disso, buscamos caracterizar qual a relação entre as funções executivas e a linguagem nesses quadros. A memória de trabalho (MT) foi a medida cognitiva mais frequentemente citada, sendo avaliada por diferentes tarefas de Span. Encontrou-se relação entre MT e discurso narrativo e conversacional, habilidades de escrita e compreensão gramatical. Outros testes cognitivos usados incluem os testes Trail Making, Wisconsin, Stroop e Verbal Fluency. Linguagem e EF têm uma relação complexa e, portanto, uma avaliação completa desses pacientes deve refletir o dinâmico processamento do funcionamento cognitivo.

Several neurological and psychiatric disorders, such as Alzheimer’s disease, primary progressive aphasia, post-stroke aphasia, traumatic brain injury and schizophrenia, can be accompanied by impairments in different language domains,^1^^-^ 7 including phonological, lexical, semantic, prosodic, discursive, syntactic aspects. These language impairments affect quality of life, autonomy, prognosis, social relationships and increase patient and caregiver burden.^8^^-^ 9 Some clinical conditions are known for having acquired language impairments, such as post-stroke aphasia, dementia, right-brain damage (RBD) and traumatic brain injury (TBI).

Alzheimer’s disease (AD) is the most common cause of dementia, responsible for 55% of all dementia cases.^10^ Even in its prodromal and early stages, it is already possible to identify changes in language abilities, mainly in the temporal characteristics of spontaneous speech, such as speech tempo, number of pauses in speech, and their length,^11^ in addition to naming disorders, impaired auditory and written comprehension, and semantic paraphasia.^12^ On the other hand, repetition abilities and articulation seem to be relatively intact.^13^ Different patterns of language impairment can be observed as the disease progresses.^14^

Aphasia can be defined as the inability to comprehend and/or formulate language because of damage to specific brain regions.^15^ Around 20% of patients who suffer a stroke are affected by aphasia.^16^ Post-stroke aphasia may occur at the phonetic, syntactic, semantic or pragmatic level of language processing. Aphasia has a heterogeneous presentation and can range from only an occasional difficulty in word-finding to losing the ability to speak, read, or write.^17^

Unlike left- brain damage, RBD does not present clear and widespread failures of language comprehension or extreme difficulty producing fluent speech. Fundamental word and sentence processing abilities are relatively unaffected by RBD.^18^ On the other hand, patients after RBD may present difficulties in discourse comprehension and higher-level language tasks related to semantic and lexical processing.^19^ In addition, they can also present limitations relating to figurative cues in language, as they tend to understand sentences from their literal meanings.^20^ Finally, most TBI patients also have relatively spared lower-level language skills, in contrast to post-aphasia patients. However, they have difficulties in more complex/high-order language tasks, such as discourse.^21^ In addition, some patients may experience difficulties interpreting non-verbal signals such as body language and emotional signals, as well as prosodic dysfunction.

For several years, language impairments were considered isolated impairments from other cognitive domains. However, recent studies have highlighted the relationship between language domains and other cognitive functions, such as mnemonic, attentional and executive,^22^^,^ 23 and their importance for therapy outcomes.^3^ For this reason, it is important to take into account cognitive performance with associations and dissociations among attention, memories, executive abilities and different linguistic components when assessing and planning interventions for patients with language disorders. Both assessment and rehabilitation are influenced by the inter-relationship of language and other cognitive domains regarding their processing.

One of the most studied cognitive abilities are the executive functions (EF), the most complex and high-order neuropsychological components responsible for deliberating control of goal-oriented actions as well as for cognitive energy distribution.^24^ In the literature, there are several established models that outline these components.^25^^-^ 28 Among the most recent theoretical proposals, the model proposed by Diamond^29^ holds there are three core EF (inhibition, working memory and cognitive flexibility). These abilities are important for different life domains, such as work and school, mental and physical health, cognitive, social, and psychological development. EF are also central to other cognitive functions, such as language.

Deficits in executive functions can result in a reduced ability to organize thought and therefore language expression, where such impairment can lead to the production of irrelevant utterances, word-finding problems, impaired sequence at the word and propositional level, tangential language and verbosity leading to problems in communication abilities.^30^ EF are necessary for a successful conversation, such as being able to retain what was said by the other person, to plan a response, and if necessary, to inhibit an inappropriate response, relying on different processes such as working memory, planning, and inhibition.^31^

Pragmatic deficits are related to difficulties in communicating effectively in interaction contexts. More specifically, these deficits and EF components of working memory, verbal planning, initiation and inhibition, switching, shifting and strategy maintenance seem to have an association.^32^ Meanwhile, several variables of conversional discourse are related to cognitive flexibility, inhibition, verbal and visuospatial planning and processing speed.^33^^-^ 36 These relationships between language and EF may manifest in patients with an acquired language disorder and can be relevant for assessment and rehabilitation programs.

The relationship between language and EF has been studied in healthy subjects. The more complex the language ability, the greater the recruitment of EF. These functions are recruited in a variety of language skills. For example, in order to tell a story, EF skills are required, such as planning and organization, otherwise the story may lack structure or important details. For auditory comprehension, it is necessary to inhibit distractions, while keeping the important information in working memory. Also, for reading comprehension, one needs to sustain attention on the text while keeping the information in working memory, integrating world knowledge with the new information acquired. Cognitive flexibility may help the reader to understand the text, even though they may not recognize a few words. These relationships also occur in individuals with different brain lesions, however, this may not take place in the most fluid way possible. The higher the pathological severity, the more EF will be necessary for language expression, in an attempt to compensate for the deficit in linguistic processing.

Although the relationship between linguistic and other cognitive dimensions has been increasingly studied, this interaction approach may be very challenging, mainly due to the fact that mnemonic, attentional, executive tasks may assess cognitive domains by means of linguistic stimuli, that is, there is underlying linguistic processing and/or production demands.^37^ Also, language batteries are already extensive and adding a complete cognitive assessment would require numerous sessions for assessment, making it difficult for speech therapists to conduct both assessments. Also, an overly long assessment process can be very stressful for the patient. An accurate diagnosis of which functions are impaired, in addition to the identification of interference of a specific deficit in other functions, will increase the specificity of treatment plans.

To the best of our knowledge, there is no other systematic review of clinical tools targeting neurocognitive assessment of EF in patients with language impairment. Therefore, we aimed to investigate which main EF components may cause the greatest impact in language disorders, as well as the relationship itself, considering the most prevalent clinical conditions associated with acquired language impairments in adults, including post-stroke aphasias, traumatic brain injury, dementias and right-brain damage. In addition, we sought to investigate which neuropsychological tests are being used to evaluate which domains.

## METHODS

For the selection of the abstracts, the Pubmed database was used together with the Prisma method for this article. The inclusion criteria for selecting the abstracts were: [1] empirical and clinical articles, [2] written in English, [3] published between 2000 and 2015, [4] with sample composed by adults with Alzheimer’s disease, traumatic brain injury, post-stroke aphasia or right- brain damaged patients [5] that had at least one executive component specifically assessed or at least a whole general executive function battery applied, and [6] discussed the relationship between language and at least one executive function. The present study also only included patients with a single brain lesion.

The keywords used were the following: executive functions OR inhibition OR inhibitory control OR interference control OR cognitive flexibility OR mental flexibility OR set shifting OR working memory OR central executive AND acquired communication disorders OR language impairment OR language disorder OR language deficits AND right-hemisphere damage OR right brain damage OR aphasia OR traumatic brain injury OR Alzheimer’s disease OR frontal temporal dementia OR dementia AND cognitive assessment OR cognitive screening OR neuropsychological assessment OR cognitive evaluation OR neuropsychological evaluation.

The exclusion criteria for the articles were only language assessment without at least one executive component (working memory, cognitive flexibility or inhibitory control) specifically assessed or at least a whole general executive function battery applied; rehabilitation and review articles; samples that included conditions other than Alzheimer’s disease, traumatic brain injury, post-stroke aphasia and right brain damaged patients.

Three independent judges analyzed 800 abstracts considering the inclusion and exclusion criteria of the present study. Initially, 28 abstracts were excluded because the related article was written in a language other than English and 33 involved children. 197 abstracts did not include one of the studied conditions (Alzheimer’s disease, traumatic brain injury, post-stroke aphasia and right brain damage). Reviews accounted for 27 dismissed abstracts. In addition, 7 rehabilitation articles were excluded. Other abstracts did not explore the relationship between language and at least one EF component and were therefore excluded. Finally, 107 articles, that had the concordance of at least 2 judges, were analyzed by the main author. However, after reading these articles, we only selected the 29 articles addressing the relationship between language and EF for discussion.

## RESULTS

The main results will be presented by pathology, mentioning the main neuropsychological tests used.

**Figure 1:**
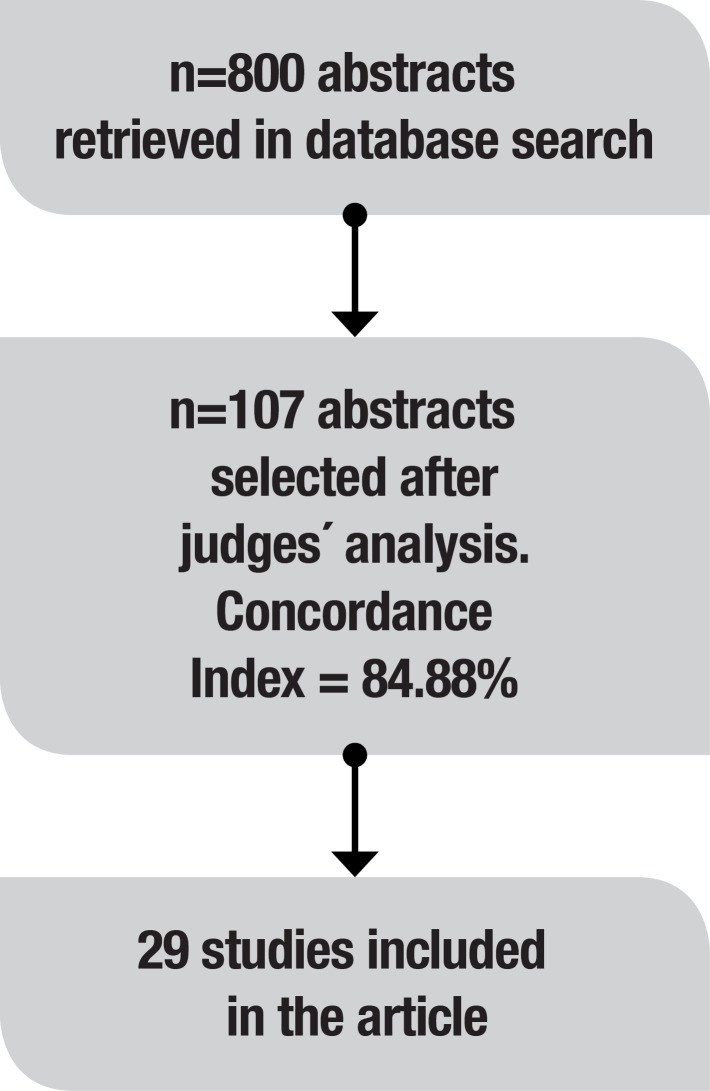
Article selection process.

In addition, the relationship between EF and language found in each article is explored. The results are presented by clinical condition because their linguistic features can vary considerably.

### Right brain damage

Only two articles with right brain damage (RBD) were selected. The first article, by McDonald,^38^ employed two verbal tests, since tools that rely on visuospatial skills are not appropriate for RBD. These two tests were the Controlled Oral Word Association Test (COWAT) for assessing generativity and rule compliance and the WAIS-R Similarities subtest (WAIR-S) for assessing verbal conceptual abilities. The second article, by Zimmermann et al.,^39^ evaluated patients with RBD by verbal fluency for three different production criteria (unconstrained, phonemic, and semantic). Performance of verbal fluency tasks seem to rely on a basic set of cognitive processes, such as sustained attention, search strategy, inhibition, and working memory.^40^

This first article sought to investigate the hypotheses that difficulties in communication reported in RBD patients would reflect a loss of executive control secondary to damage to the frontal systems of the brain or their connections.^38^ However, EF were not significantly associated with pragmatic competence in production or reception in this study. The reasons explaining why the executive dysfunction was unable to predict general pragmatic performance is unclear but could be related with the unilateral nature of the brain damage experienced. This study also showed that, for the executive impairments to disrupt pragmatic language skills, they may need to be particularly pervasive, or perhaps of a particular kind.^38^

Zimmerman et al.,^39^ found that a larger number of patients with RBD had impairments in semantic verbal fluency, more associated with semantic memory retrieval than with phonemic or unconstrained verbal fluencies. A greater number of patients were detected using a longer task, when compared to a shorter version, which indicate more difficulty maintaining lexical search over time than actual executive impairments. Therefore, RBD patients seem to rely less on EF during verbal fluency tasks.^39^

### Traumatic brain injury

Working memory was the EF component most studied in TBI articles.^34^^,^ 41 ^-^ 46 The three main EF tests used were the Trail Making Test,^42^^,^ 43 ^,^ 45 ^,^ 47 ^,^ 48 Span^34^^,^ 41 ^-^ 43 and Stroop^45^^,^ 47 ^,^ 48 tasks. The Trail Making test was used to assess cognitive flexibility, a complex attention and planning. Articles did not specify for which EF component the Stroop task was used. Finally, Span tasks were used to assess working memory abilities.

Studies that sought to investigate the relationship between EF and discourse production found modest significant correlations between measures of WM and narrative discourse.^49^ These findings suggest that the story retelling task placed a greater demand on WM by requiring information processing and temporary storage of the information necessary for accurate retelling.^49^ However, Digits Backwards, a task usually used to assess WM, did not prove to be a significant predictor of pauses produced between clauses between speakers with TBI.^43^ On the other hand, the test of Likeness-Differences provides a verbal measure of EF and was able to predict the number of mazes produced per utterance. These findings suggest that microlinguistic deficits can be explained by deficits in the way individuals with TBI recruit and control attention for sentence planning.^43^

In addition, it was found that the poorer the performance on the executive tests, the more communication difficulties were present.^50^ Several specific items of a discursive test were found to be correlated with EF measures among TBI patients in the acute care phrase. Early conversational discourse in the acute care phase post-TBI is significantly related to WM, cognitive flexibility, divided attention, and initiation abilities.^36^ However, only a weak relationship was found between WM measures and inference production. Also, the TBI and control groups did not differ on the measure of proportion of explanations and predictions that relied on the maintenance of information in WM as the source.^46^ Thus, it is unlikely that group differences in WM can explain the poorer narrative comprehension and tendency to focus more on local, sentence by sentence, detail in the TBI group.^46^

**Table 1:** Articles included in review.

Reference	Aim	Sample	Language assessment	EF assessment
Mcdonald, 2000	Investigate two alternative explanations for the cognitive bases of linguistic dimensions of RBD.	Right Hemisphere CVA infarction or hemorrhage	A pragmatic battery representing a range of tasks assessing both pragmatic production and comprehension.	Controlled Oral Word Association Test (COWAT); WAIS-R Similarities subtest
Zimmermann et al., 2013	Verify dissociations in the performance of verbal fluency tasks with different production criteria and duration following vascular right-hemisphere damage	Vascular right-Hemisphere Damage	Unconstrained, phonemic, and semantic fluencies from the Montreal Communication Evaluation Battery (1 and 2 minutes fluency tasks)	
Youse et Coelho, 2005	Investigate the relationship between working memory and narrative discourse production in individuals with closed head injury.	Closed Head Injury	Story retelling (The bear and the fly) and story generation (Norman Rockwell painting, The Runaway).	Digit Span, Logical memory and Associative Learning tasks from WSM
Sainson, Barat et Aguert, 2014	Improve assessment of the non-verbal as well as verbal aspects of the communication disorders observed in TBI subjects. Also, to demonstrate validity of the grid for linguistic analysis (GALI) tool as a means of measuring interactive skills in a given population.	Severe Traumatic Brain injury	Grid for linguistic analysis (GALI) of free conversational interchange	Tower of London; Stroop; Wisconsin Card Sorting Test (in the form modified by Nelson); Trail-Making Test; Cube construction and picture arrangement subtests from WAIS-R battery; Six Elements test
LeBlanc et al., 2014	Examine the relationship between conversational discourse and performance on other neuropsychological and language tests to explore the validity of this test as compared with other tests measuring cognition (attention, memory, and mental flexibility) and language (naming, fluency, and verbal reasoning). Moreover, analyze the relationship between conversational discourse and outcome at discharge from acute care.	Traumatic Brain injury	D-MEC; short-form of the Boston Naming Test; Verbal absurdities sub-test of the Detroit Test of Learning Aptitude; Verbal fluency	Digit Span subtest from the WMS-III battery; Trail-Making Test; Hopkins Verbal Learning Test; Verbal fluency
Coelho et al., 2013	Examine discourse performance of a large group of individuals with penetrating head injury. Performance was also compared across 6 subgroups of PHI based on lesion locale. A preliminary model of discourse production following PHI was proposed and tested.	Penetrating Head Injury	Sentence production, cohesive adequacy, coherence, story grammar, completeness, and reliability based on the 16-frame picture story ‘Old McDonald Had an Apartment House’	Sorting Test composite scaled score from the Delis-Kaplan Executive Function System Test; Letter-number sequencing and Spatial span tasks from the WSM-III
Peach, 2013	Investigate the cognitive basis for microlinguistic deficits in individuals with TBI.	Severe Traumatic Brain injury	WAB ‘‘Picnic Scene’’; Neurosensory Center Comprehensive Examination for Aphasia; Sentence Repetition (simple recall); Boston Naming Test	Halstead-Reitan; Trail-Making part B; Wechsler Memory Span; WMS Digit Span Backwards; NCCEA Word Fluency; Detroit Tests of Learning Aptitude Likenesses and Differences; Raven’s Coloured Progressive Matrices
Douglas, 2010	Explore the behavioral nature of pragmatic impairment following severe TBI and to evaluate the contribution of executive skills to the experience of pragmatic difficulties after TBI.	Severe Traumatic Brain Injury	La Trobe Communication Questionnaire (LCQ)	COWAT or FAS Verbal Fluency tasks; RAVLT; Speed of Comprehension - subtest SCOLP
Rousseaux, Vérigneaux et Koslowski, 2010	Analyze conversation at the rehabilitation and chronic phase post-TBI and to define the main mechanisms of verbal and non-verbal communication disorders and relationship with other cognitive difficulties.	Severe Traumatic Brain Injury	Lille communication test; Scene Description and Picture Naming subtests from Montreal-Toulouse protocol of aphasia examination	Stroop test; Trail-making test (TMT A and B); Verbal fluency categorical recall (animals, 1 min)
Thomas-Antérion et al., 2009	Investigate semantic memory in brain-injured patients.	Traumatic Brain injury and Stroke	New word questionnaire	16-item Free and cued selective reminding test; Digit symbol and vocabulary subtests from WAIS-III battery; Trail-making test parts A and B; Stroop test; two-minute verbal fluency (animal category and letter ‘‘p’’); DO80 picture naming task
Schmitter-Edgecombe et Bales, 2005	Examine the content of information available to working memory during narrative comprehension in a TBI population using a think-aloud method.	Severe Traumatic Brain Injury	Participants were presented with two narratives that each contained 18 sentences. Both stories provided ample opportunity for participants to draw inferences while reading. The final sentence of each story was also designed to elicit recall of information presented earlier in the story. Five true/false questions, which inquired about simple factual knowledge that was explicitly stated in the narratives, were also derived for each story.	Working Memory Span and Letter Number Sequencing subtests from the WAIS-III; Wisconsin Card Sorting Test; Stroop Color and Word test; Verbal fluency from COWAT; Symbol Digit Modalities Test
Small, 2000	Investigate the role of two additional factors known to contribute to the complexity of sentence processing: canonicity of thematic role assignment and branching direction of relative clauses. In addition, the relationship between sentence-repetition performance and processing resource capacity is examined.	Mild-to moderate-stage Alzheimer’s Disease	Phrase repetition variation is measured for six types of sentences varying for three dimensions of syntactic complexity: canonicity of thematic role assignment, branching direction of embedded relative clauses, and number of verbs (or propositions) in the sentence	Digit span; Days of the week and the months of the year backwards
Herbert et al., 2014	Investigate the specific pattern of verbal fluency performance in cerebral small vessel disease (SVD), and compare this with Alzheimer’s disease (AD).	Small vessel disease and Alzheimer’s disease	Verbal Fluency	The Brief Memory and Executive Test (BMET); Mini; The National Adult Reading Test-Revised (NART-R)
Wardlow, Ivanova et Gollan, 2014	Examine how AD changes speakers’ ability to take their listeners’ perspective in a verbal communication task and links this performance to processing models of both perspective taking itself and, more broadly, to cognitive decline in AD.	Mild-to-Moderate Alzheimer’s disease	Referential communication task modeled after Wardlow Lane and Ferreira (2008); Verbal fluencies; Boston Naming Test	Forward and Backward Digit Span; Trail-Making Tests A and B; Flanker Task; Hayling Task
Yoon et al., 2011	Explore the diverse error patterns manifested in writing single syllables in Korean patients with early onset Alzheimer’s disease	Early onset Alzheimer’s disease	Korean version of the Western Aphasia Battery (WAB) - spontaneous speech, auditory comprehension, repetition, naming, reading, and writing subtests	Seoul Neuropsychological Screening Battery (SNSB); Digit span (forward and backward); Rey-Osterrieth Complex Figure Test; Seoul Verbal Learning Test; Phonemic and semantic from COWAT; Stroop Test.
Stoford et al., 2007	Examine patterns of memory impairment in a large cohort of AD patients, with particular attention to the relationship between working and long-term declarative memory.	Mild-to-Moderate Alzheimer’s disease	Verbal recall; Categorical and Phonemic Verbal fluencies (1 minute)	Simplified version of the Brown- Peterson paradigm
Feyereisen, Berrewarts et Hupet, 2007	Study to what extent persons suffering from AD can benefit from shared experience through trial repetition to achieve common reference.	Mild Alzheimer’s disease	Picture description task; Referential communication task	Categorical and Phonemic Verbal Fluency (2 minutes); Hayling Test and Stroop Test
Papagano et al., 2003	Examine the relation between idiomatic and literal language in Alzheimer’s disease patients and the role of executive functions in idiom comprehension.	Mild Alzheimer’s disease	Sentence-to-picture matching task; Literal sentence comprehension	Pencil-and-paper dual task (digit span and tracking)
Waters et Caplan, 2002	Examine the relationship between working memory capacity and the ability to structure sentences syntactically online.	Mild-to-Moderate Alzheimer’s disease	Online auditory sentence comprehension test	Alphabet Span task; Backward Digit Span task; Sentence Span
Small, Kemper et Lyons, 2000	Examine the effects of grammatical and extragrammatical variables on sentence production in AD.	Mild-to-moderate Alzheimer’s disease	Six sentence types (Active, Passive, Object-Subject, Object-Object, Subject-Subject, and Subject-Object)	Forward and Backward Digit Span; Days of week/months of year backwards
Caplan, Michaud et Hufford, 2013	Examine the relation between mechanisms that support short-term memory performance and syntactic comprehension in patients with aphasia.	Post-stroke aphasia	Object manipulation task; Sentence–picture matching with uninterrupted auditory presentation; Sentence–picture matching with auditory self-paced (auditory moving window) presentation	Alphabet Span; Backwards Digit Span; Subtract-2 Span; Sentence Span.
Ivanova et Hallowell, 2014	Develop and test the concurrent validity of a WM complex span task suited for individuals with aphasia and establish the psychometric properties of associated performance measures.	Post-stroke aphasia	Aphasia Quotient components of the Western Aphasia Battery-Revised	Traditional listening span; Modified listening span task - Length and complexity of sentences were manipulated separately, creating conditions with: (a) short and simple; (b) short and complex; (c) long and simple; and (d) long and complex sentences
Mayer et Murray, 2012	Examine the feasibility, reliability, and internal consistency of an n-back task for evaluating WM in aphasia, then explore the influence of domain-general (WM load, reaction time, age) and domain-specific (language) factors.	Aphasia after a left hemisphere lesion	Western Aphasia Battery; Confrontation-naming task for the linguistic stimuli	3 N-back test
Murray, 2012	Further elucidate the relationship between cognition and aphasia, with a focus on attention.	Post-stroke aphasia	Aphasia Diagnostic Profile; American Speech-Language Hearing Association Function Assessment of Communication Skills for Adults	Test of everyday attention; backward memory span; Tompkins et al. working memory protocol; Ruff Figural Fluency Test
Potagas, Kassemilis et Evdokimidis, 2011	Investigate short-term memory and working memory deficits in aphasics in relation to the severity of their language impairment	Post-stroke aphasia	Auditory sentence comprehension, and oral expression subtests from the Boston Diagnostic Aphasia Examination–Short Form (BDAE-SF) adapted in Greek	Digits backward subtest from WAIS-III battery
Knibb et al., 2009	Provide a detailed quantitative description of conversational speech, along with cognitive testing and visual rating of structural brain imaging. Additionally, to examine which, if any, features were consistently present throughout the group. As well as looking for sub-syndromic associations between these features.	Progressive non-fluent aphasia	Semi-structured conversation of 15–20 minutes. The following quantitative measures were calculated: speech rate, phrase length, and syntactic complexity.	Graded Naming test; Test for Reception of Grammar (TROG); the 64-item Camel and Cactus Test of non-verbal semantic ability; the 64-item Cambridge spoken word-picture matching test; the Wisconsin Card Sorting test
Seniów, Litwin, Lesniak, 2009	Determine whether post-stroke aphasia is associated with impairments of visuo-spatial working memory and abstract thinking and whether these deficits adversely affected language recovery.	Post-stroke aphasia	Visual Confrontation Naming, Body-part Naming, Repetition of Words, Repetition of Phrases and Sentences, Word Discrimination, Body part Identification, Commands, and Complex Ideational Material subtest from the Boston Diagnostic Aphasia Examination	Standard Progressive Matrices; Benton Visual Retention Test
Fridkisson et al., 2006	Investigate the relationship between functional communication and executive function ability in aphasia.	Post-stroke aphasia	Speech-Language Hearing Association Functional Assessment of Communication Skills for Adults; Bedside Evaluation Screening Test (2nd ed.)	Color Trails Test; Wisconsin Card Sorting Test-64 Card Version
Grossman et Moore, 2005	Determine how grammatical, single word meaning, and working memory factors contribute to longitudinal decline of sentence comprehension in primary progressive aphasia.	Primary progressive non-fluent aphasia	Sentence Comprehension Task with different complexity loads	Digit Span

Approximately one third of variability in the pragmatic problems reported was accounted for by measures of EF. Performance on a phonemic verbal fluency task was the only task able to predict pragmatic impairment.^44^ There was also a pattern of association between the ability to maintain information over time, performance and pragmatic competence. These findings suggest that impaired storage and retrieval processes can contribute to problems of relevance including topic management in conversation.^44^ EF integrity seems to be generally necessary for a successful conversational interchange to be maintained. However, unlike verbal communication, non-verbal communication did not seem to be related to any EF test.^48^

In a study with penetrating head injury, IQ was highly correlated with the cognitive variables of EF, WM and immediate memory, and by having these highly correlated variables as predictors of discourse measures in the model, the paths between IQ and cognitive ability were considered to be potentially redundant. IQ seems to account for cognitive ability, story completeness, and story grammar.^34^ A group with penetrating head injury had impairment in WM and immediate memory, possibly explaining the patients’ difficulty with completeness and story grammar, which may reflect their difficulty developing a mental representation of the story they saw and were asked to retell. The retelling was incomplete and disorganized, because of an inability to process the story, a lack of foundation or inefficient mapping.^34^

Finally, a study that sought to investigate semantic memory in TBI patients based on their capacity to learn new words that had recently entered the French language found correlation between the processing of new words from 2006 to 2007 and patients’ performance in the letter fluency and vocabulary tests.^45^ The difficulty in defining new words is probably related to difficulties retrieving lexical knowledge and in EF.^45^

### Dementia

The most used EF test for assessing patients with dementia was the different modalities of digit spans used to assess WM abilities. For evaluating general EF performance, the phonemic and semantic verbal fluencies and the Trail Making Test were used. The Hayling Test was used in two studies for assessing inhibitory control.

Overall fluency impairment in small vessels disease seemed to be related to EF and processing speed deficits, common in this condition. These patients have relatively preserved semantic memory, so do not have an added disadvantage in semantic fluency tasks.^51^ On the other hand, the AD group presented a discrepancy between phonological and semantic fluency, which may be explained by the greater reliance of semantic fluency tasks on semantic memory, which is degraded in AD.^51^

Regarding sentence production, there seems to be a strong correlation between performance on WM tests and the sentence-repetition task. The strongest correlations were found in the sentence types with embedded relative clauses. However, correlations that involved Active and Passive sentences were less robust.^52^ Tasks that demanded more WM had the strongest correlations.^52^ However, a reduction in WM capacity was not associated with a decreased ability to devote processing resources to syntactic analysis and to the use of syntax to determine sentence meaning.^53^ In addition, strong relationships were not evident between the reverential communication task scores and inhibitory control mechanisms in the early stage of AD.^54^ On the other hand, familiar idioms need suppression of the literal interpretation in order to for idiomatic meaning to be derived. This suppression in mediated by the central executive which may be impaired in AD patients, therefore the inhibition of the literal meaning is not efficient and the figurative meaning does not get sufficient activation.^56^ Also, a close relationship between WM and language was identified, evidenced by the fact that complex skills placing demands on working memory (i.e., digit reversal, spelling, and calculation) did not place demands on the memory or the executive factor, but rather on a general language factor.^55^

Perspective-taking performance was related with reasoning skills, the integrity of semantic memory, management of attention and response conflict.^56^ Regarding writing skills, the number of correct responses in a writing task was related with working memory, immediate recall, inhibition and planning.^57^ Finally, metaphor comprehension seems to be more related to EF, than language skills themselves.^55^

### Aphasia

WM appears to be the most studied EF in aphasia patients, but there were some studies with different kinds of attentional systems and the EF of inhibition, planning, cognitive flexibility, working memory, sustained attention, perceptual tracking, and graphomotor skills. WM was measured using the backward visual memory span subtest of the Wechsler Memory Scale and the digits backwards of the WAIS-III, in addition to other spans tasks such as Alphabet Span, Subtract-2 Span, and Sentence Span.

Better scores on short-term memory tests were associated with both experimental and baseline sentences. The dissociations found between normal performance in sentence comprehension and below normal performance on short-term memory (STM) indicates that normal STM is not necessary needed to support either parsing and interpretation *per se* or the use of STM in mapping interpretations onto operations required to perform tasks.^58^

Passive sentences were more difficult to understand compared to active (as indicated by lower processing scores), and longer sentences were more difficult than shorter ones. These results were expected, given that comprehension deficits are characteristic of aphasia, and that individuals with aphasia tend to have difficulties understanding sentences with noncanonical thematic role orders such as passives. Neither complexity nor length of sentence by itself impacted recall.^59^ The ability to switch between processing and storage in the WM span task is the primary influence on WM capacity indices, not the difficulty of the task or characteristics of the linguistic stimuli.^59^

Patients with aphasia present impairments in visual-spatial WM and difficulties in generalization and abstraction on non-verbal material.^60^ After a rehabilitation program, an improvement was observed in two important language functions, naming and comprehension, that was associated with patients’ baseline non-verbal visual-spatial working memory.^60^

A correlation with grammatical comprehension deficit and limited WM capacity can also be seen in progressive non-fluent Aphasia.^61^ However, this correlation seems to be specific for grammatically complex sentences, given there were no significant correlations with grammatically simple sentences.^61^ Aphasia severity symptoms were associated with patient performance on WM measures, for both verbal and spatial WM scores.^62^

A correlational and regression analyses showed significant relationships between participants’ attention deficits and their language and communication performance. The more complex attention skills had a stronger correlation with language and communication. It also was found that attention allocation difficulties negatively affected auditory comprehension and spoken language.^63^

For some aphasic patients, executive functioning may be a better indicator of functional communication ability when compared to language impairment.^31^ Decreased fluency was also associated with a decline in functional communication ability. These results may indicate that a decrease in fluency is a greater barrier to functional communication than impaired auditory comprehension.^31^

However, another study evaluating conversional abilities found no correlation between cognitive impairments and the severity of spoken language impairments.^64^ Another study also failed to find an association between language and WM skills. These findings could be explained by the fact that the simple language tasks requiring basic linguistic operations and direct retrieval of lexical items from the semantic system, such as naming of objects, single word comprehension, single word repetition, and production/comprehension of short and simple sentences, do not rely heavily on WM.^23^ Perhaps, with more complex language tasks, particularly tasks targeting receptive language abilities, an association can be found between WM and language measures.^23^

## DISCUSSION

Language skills are essential for successful social interaction in different domains such as personal relationships and the work environment.^65^ Linguistic impairments may be a reflection of deficits between cognition and linguistic processing, rather than a specific difficulty of language.^66^

Considering the importance of further elucidating the relationship of executive functioning and language impairments, the aim of this article was to define which EF components were the most present when assessing patients with language disorder and which neuropsychological tests were chosen for their assessment. Also, the study sought to investigate the relationship between EF and language in the most prevalent acquired communication disorders among adults.

WM was the cognitive measure most frequently chosen in the articles of this review. It was present in studies of TBI,^49^ dementia^56^ and aphasia.^58^ Different span tasks were used to assess WM, such as alphabet span,^58^ sentence span^58^ and a modified version span.^59^ Digit span was the most commonly used cognitive test to evaluate WM, present in several studies.^43^^,^ 45 ^,^ 49 ^,^ 52 ^,^ 53 ^,^ 56 ^,^ 57 ^,^ 67

WM has an impact on several language domain, such as narrative discourse,^49^ completeness and story grammar,^34^ conversational discourse,^42^ inference production,^46^ sentence repetition,^52^ number of correct answers in a writing task,^57^ improvement in naming and comprehension after rehabilitation^60^ and grammatical comprehension.^61^ However, no relationship was found between WM measures and pauses produced between clauses^43^ or in online syntactic processing.^53^

Other cognitive tests used in several articles included the Trail Making Test,^36^^,^ 43 ^,^ 45 ^,^ 48 ^,^ 50 ^,^ 56 the Wisconsin Test^31^^,^ 50 ^,^ 46 ^,^ 68) and the Stroop test.^48^^,^ 50 ^,^ 45 ^,^ 54 ^,^ 57 The Trail Making Test is a valuable tool for assessing cognitive flexibility, working memory, set-shifting and inhibitory abilities.^69^ The word-color Stroop test, however, measures attention, cognitive flexibility, inhibition and information processing speed.^70^ The Wisconsin Test is a widely used neuropsychological task for the assessment of higher-order cognitive functioning, and test performance is associated with abstract reasoning, strategic planning, organized searching, and impulse control abilities.^70^ However, it has been heavily criticized for being too general a tool for EF measurement.

Interestingly, one of the most used paradigms for both language and other neuropsychological domain assessments was verbal fluency (VF), in both its modalities, categorical and phonemic. It was present in studies of RBD,^39^ dementia^51^^,^ 55 ^-^ 57 ^,^ 71 and TBI^43^^-^ 46 ^,^ 48 ^,^ 67 results were compared to findings on tests of memory, mental flexibility, confrontation naming, semantic and letter category naming, verbal reasoning, and to scores on the Montreal Cognitive Assessment. The relationship to outcome as measured with the Disability Rating Scale (DRS. VF tasks are often used in clinical and research practice, and their wide-spread use is partially due to their validity for both verbal and executive abilities. It is considered an effective screening instrument for general verbal functioning and its validity for assessing executive control is well established.^72^

Cognitive assessment can be especially challenging in patients suffering from linguistic deficits given they present problems in comprehension. Those problems may result in misleading scores on cognitive tests, because when patients are unable to understand what they have been asked to do, their performance will be affected.^62^ Therefore, it is important to devise cognitive screening tests and batteries based on the main executive functions and in the most used neuropsychological tests specifically designed for language impairment.

This kind of battery could be useful for speech language therapeutic use, since it would be a rapid way of identifying the EF deficits that can impact communication. On the other hand, it is also necessary to draw on the further understanding of the relationship between language and EF to devise tests and evidence-based interventions designed for speech language therapists. Furthermore, this could also raise awareness of neurologists and neuropsychologists regarding the importance of assessing communication disorders when assessing cognitive functions.

Several EF such as planning, working memory, accurate self-monitoring, and judgment are necessary skills for patients to engage in, and benefit from speech language therapy.^73^ They play an important role for rehabilitation because they underpin several complex behaviors needed for functional independence and social integration.^74^ The understanding of the relationship between EF and language could help design optimal interventions for patients suffering with language disorders. Therefore, an important step for developing communication strategies by health care professionals, caregivers and patients themselves is to try to identify the nature of the problems that are causing communication breakdowns.

As shown throughout this article, language and EF have a complex relationship, therefore a complete assessment of patients with an acquired language disorder should reflect the dynamic processing of cognitive brain functions, as opposed to a compartmentalized one. Therefore, it is essential that clinicians, language speech therapists and neuropsychologists work as a team to offer the best healthcare service possible.

Some limitations of this study relate to the fact that the articles selected have very heterogeneous samples when compared, which can have an impact on the results. Also, many different types of linguistic processes were included, therefore the relationship of EF with which aspect of language processing was unclear. Another limitation concerns the use of only one research engine for the articles search.

EF appears to play a crucial role in comprehension and production of linguistic macrostructures^33^ and microstructures.^43^ However, the extent to which EF interferes with several language domains is still unclear and needs to be further elucidated. Of the main benefits of considering the relationship of language and EF subprocess, we highlight the accuracy of rehabilitation planning. During rehabilitation, for example, clinicians could use this relationship to improve both processes. Some linguistic symptoms are strongly related to a dysexecutive syndrome, such as perseveration, off-target verbosity, stereotype, and could benefit from this vision.

More studies should be conducted to better understand the relationship between language and EF and its influence on speech language therapy. Further, future studies should explore the influence of other components that could interfere in this relationship. It should be considered that executive functioning it not only responsible for basic cognitive processes, but also for complex systems that integrate these abilities.^74^
